# Cold adaptation, aging, and Korean women divers haenyeo

**DOI:** 10.1186/s40101-017-0146-6

**Published:** 2017-08-08

**Authors:** Joo-Young Lee, Joonhee Park, Siyeon Kim

**Affiliations:** 1 Seoul National University, Bld.222-#306, 1 Gwanak-ro, Gwanak-gu, Seoul, 08826 South Korea; 2 Seoul National University, Bld.222-#332, 1 Gwanak-ro, Gwanak-gu, Seoul, 08826 South Korea

**Keywords:** Haenyeo, Aging, Cold adaptation, Cotton bathing suits, Wetsuits, Cold tolerance, Heat tolerance, Breath-hold diving, Cross-adaptation

## Abstract

**Background:**

We have been studying the thermoregulatory responses of Korean breath-hold women divers, called *haenyeo*, in terms of aging and cold adaptation. During the 1960s to the 1980s, haenyeos received attention from environmental physiologists due to their unique ability to endure cold water while wearing only a thin cotton bathing suit. However, their overall cold-adaptive traits have disappeared since they began to wear wetsuits and research has waned since the 1980s. For social and economic reasons, the number of haenyeos rapidly decreased to 4005 in 2015 from 14,143 in 1970 and the average age of haenyeos is about 75 years old at present.

**Methods:**

For the past several years, we revisited and explored older haenyeos in terms of environmental physiology, beginning with questionnaire and field studies and later advancing to thermal tolerance tests in conjunction with cutaneous thermal threshold tests in a climate chamber. As control group counterparts, older non-diving females and young non-diving females were compared with older haenyeos in the controlled experiments.

**Results:**

Our findings were that older haenyeos still retain local cold tolerance on the extremities despite their aging. Finger cold tests supported more superior local cold tolerance for older haenyeos than for older non-diving females. However, thermal perception in cold reflected aging effects rather than local cold acclimatization. An interesting finding was the possibility of positive cross-adaptation which might be supported by greater heat tolerance and cutaneous warm perception thresholds of older haenyeos who adapted to cold water.

**Conclusions:**

It was known that cold-adaptive traits of haenyeos disappeared, but we confirmed that cold-adaptive traits are still retained on the face and hands which could be interpreted by a mode switch to local adaptation from the overall adaptation to cold. Further studies on cross-adaptation between chronic cold stress and heat tolerance are needed.

## Background

In 2016, the culture of Jeju haenyeo, Korean breath-hold women divers, was inscribed on the representative list of the intangible cultural heritage of humanity of the United Nations Educational, Scientific and Cultural Organization (UNESCO). The word *haenyeo* literally means “sea women” in Korean and corresponding to *ama* in Japanese. During the nineteenth century, 22% of the entire female population in Jeju were haenyeos [1]. Until the early 1980s, haenyeos were known as being more tolerable to cold water than other divers due to their diving practices in winter. Sea water temperature in winter is on average 13–14 °C at Jeju island [2] and haenyeos had dived even in the middle of winter wearing only thin cotton bathing suits until the late 1970s [3]. Because of their particular diving practices with thin cotton or thick wet diving suits, professor Suk-Ki Hong and his colleagues investigated young haenyeos’ cold acclimatization and deacclimatization in terms of environmental physiology in the 1960s to the 1980s. One of the classic studies by Hong’s group found that the cold-adaptive properties of haenyeos disappeared as they began to wear new wetsuits instead of traditional cotton bathing suits in the mid-1970s [4]. Since then, investigations on haenyeos’ thermoregulatory responses to cold have waned.

The number of Jeju haenyeos, who are currently working under the sea, rapidly decreased to 4005 in 2015 from 14,143 in 1970 [5]. At present, 88% of haenyeos are over their sixties. Moreover, 57% of the 4005 haenyeos are in their seventies or older [5]. The older haenyeos have been diving more than 50 years since their teens. Haenyeos’ whole body used to be exposed to severe cold stress in their youth because they wore only thin cotton swimsuits at that time. Now, their body is insulated by thick wetsuits but the face and hands are still exposed to cold stress [6]. In other words, wetsuits protected older haenyeos from severe cold stress in winter but induced local exposure to mild cold over the face and hands because of prolonged diving hours [6]. Water has a much higher thermal conductivity than air (0.58 vs. 0.025 W/mK), and heat conductance from unprotected skin can be more than dozens of times greater in water than in air at the same temperature. For this reason, fishermen, divers, and swimmers who frequently immerse a part or all of their body in water can experience considerable body heat loss, even when the water temperature is only mildly cool [7]. In this regard, we hypothesized that the mode of cold adaptation for haenyeos would switch over to local body adaptation from the overall body adaptation in line with the change to thick wetsuits from thin cotton bathing suits. Further, we hypothesized that aging would be an influential factor on the cold-adaptive traits of the body extremities for older haenyeos. Lastly, we explored the possibility of cross-adaptation between chronic cold stress and responses to heat. With these hypotheses, we investigated older haenyeos’ physiological and behavioral thermoregulation to general and local thermal stresses for the past several years. In particular, we explored cutaneous thermal perception thresholds triggering autonomic and behavioral responses as well as thermal effector responses. In this review, we introduce our recent works with older haenyeos along with historical contribution by Hong’s group. It will be worthwhile to revisit the haenyeo culture and introduce findings on the thermophysiological changes of older haenyeos.

## General characteristics in diving practices of haenyeos

The official number of Jeju haenyeos was 26,248 in 1962 but only 4005 in 2015 [5]. The age distributions of haenyeos in the 1970s was 31% for less than 30 years old and 55% for 30–49 years old, but presently, about 88% of all haenyeos are over 60 [5]. The present older haenyeos began diving at the approximate age of 8 to 15 and have continued to dive for over 50 years. Most haenyeos are classified into three classes according to their diving skill. Class 1 is able to dive deeper than about 16 m in depth with 2-min breath-holding; class 2, about 13 m in depth; and class 3, above 13 m [3]. Haenyeos, who wore cotton swimsuits, engaged in diving for periods of 1 to 5 h daily depending on seasons and tide time. Hong and colleagues [8] reported that haenyeos made 113–138 dives a day and stayed in the water a total of 170–200 min a day, of which only 52–63 min were spent diving submerged, and the remaining time was spent at the water surface. In winter, a typical dive lasted about 60 s and consisted of a 30-s dive followed by a 30-s rest interval. Haenyeos worked an average of 28 days a month from March to August and 15 days a month during the winter season while wearing the traditional cotton bathing suit [3]. The total time spent submerged for haenyeos wearing the cotton swim suit was about 30 min in winter and about 40 to 60 min from Spring to early Autumn [1]. However, the diving shifts were prolonged due to the wetsuits from 70 to 180 min in summer and 10 to 120 min in winter compared to those when wearing the cotton bathing suits [4].

**Fig. 1:**
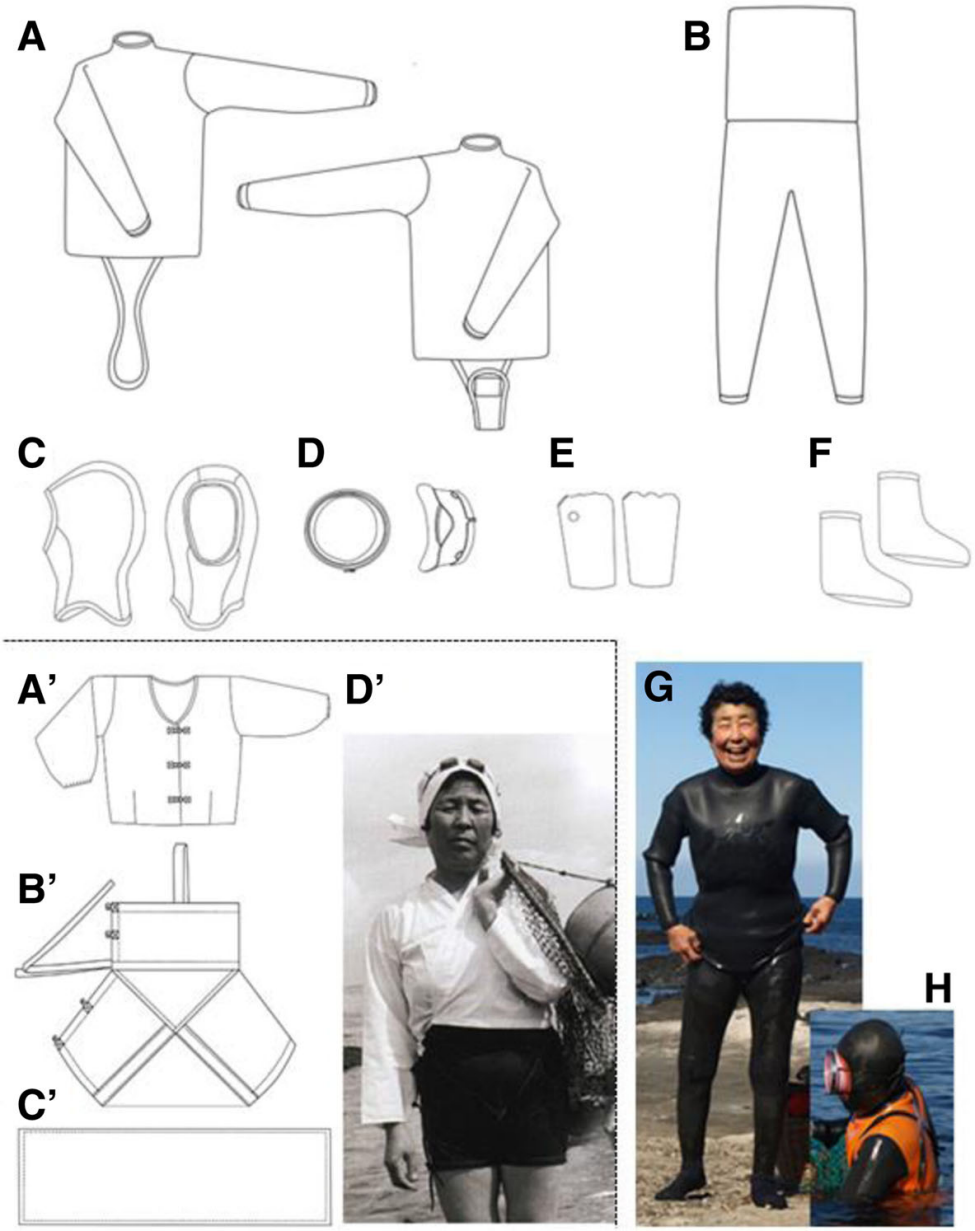
Wetsuit currently worn by haenyeos (**A**–**H)** and traditional cotton suit worn until the 1970s (**A**'–**D**'). Adapted from Lee et al. [3]

Haenyeos had originally worn a cotton bathing suit in white or black (100% cotton, about 0.5 mm thick) until wetsuits were supplied. The traditional cotton bathing suit consisted of *so-jung-ee* (bathing suit), *juck-sam* (upper shirt), and *mul-su-gun* (a hood) without any goggles (A´ to D´ Fig. 1). A to H in Fig. 1 show the wetsuits currently worn by older haenyeos. As a result of the change to wetsuits from the cotton suits, haenyeos were no longer exposed to the severe cold water [9]. Wetsuits certainly made the work hours of haenyeos longer than before. Before wearing the wetsuits, daily diving hours in winter were rarely over an hour, whereas the daily work hour increased more than an hour after the introduction of the wetsuits [10]. At present, several thicknesses of wetsuits are supplied for haenyeos: 4 mm in thickness for summer, 5 mm for spring and autumn, and 6–7 mm for winter [1].

Although cold strain during winter diving is reduced, haenyeos are typically experienced with the following occupationally related diseases: headache, hearing-impairment, otitis media, stomach disorder, skin irritation, and musculoskeletal symptoms. In particular, hearing-impairment is common for older haenyeos. Because of wetsuits and flippers, haenyeos began diving deeper and longer compared to the days of wearing thin cotton swim suits without flippers, which induced the injuries of tympanic membrane and hearing-impairment [3]. Moreover, haenyeos skip their meal before diving so as to prevent from throwing up the meal during their hand-standing-diving position and from defecating in the sea while working for several hours [11]. On the other hand, Bae and colleagues [12] discussed that the prolonged cold-water immersion of haenyeos might induce a decrease in muscle fiber size and an increase in capillarity in human skeletal muscles.

## General responses to cold

During exposure to cold stress, cutaneous cold receptors are activated to initiate the reflexes involved in conserving heat, which is accomplished through cutaneous vasoconstriction. The human body responds by decreasing heat loss through cutaneous vasoconstriction and piloerection. It is known that in a cold environment, most of the venous return from the arms and legs is through the deep venae comitantes that receive heat from blood flowing through the arteries, thereby minimizing heat loss. When body extremities such as the fingers, toes, or earlobes are exposed to severe cold, the regions show cold-induced vasodilation (CIVD). The CIVD reaction varies considerably among individuals and is the essential factor which determines individual differences of local cold tolerance. Under continuous or severe cold stress at a certain level, the human body responds by increasing heat production through shivering or non-shivering thermogenesis (NST), called cold-induced thermogenesis. Shivering is the involuntary muscle activation, and most energy in shivering is transformed into heat. An increase in metabolism without muscular movement is called NST, which is a more efficient mechanism than shivering for increasing heat production in the cold because the muscular tremor of shivering increases convective heat losses from the body surface. Noradrenaline and thyroxine are hormones that play significant roles in the development of NST [13]. One particular mechanism for NST is that concerned with brown adipose tissue (BAT). Animals exposed to cold stress exhibit a marked increase in BAT. Decreases in body temperature of animals exposed to −25 °C for 3 h were inversely related to the mass of BAT [14]. In recent years, a group of researchers has shown that BAT is present in human adults, showing the relation to NST [15–17]. A 10-day cold acclimation protocol in humans increased BAT activity along with an increase in NST [16]. Cold-induced thermogenesis through both shivering and BAT is likely to be involved with mitochondrial uncoupling which is activated by the sympathetic nervous system [17]. On the other hand, local exposure to cold can elicit cold-adaptive traits. Repeated local cooling (forearm cooling using 5 °C water cooling pads, eight times for 3 weeks and 70-min cooling per time) affects oxidative metabolism in skeletal muscle metabolism, and this adaptation may facilitate the performance of the muscle in cold [18].

## Dr. Hong’s contribution to thermal physiology of haenyeos

### Cold acclimatization and haenyeos of the past

From the 1940s to the 1970s, Australian aborigines, Kalahari Bushmen, Alacaluf Indians, Andean Indians, Eskimos, Nomadic Lapps, Japanese ama, and Korean haenyeos have been explored in terms of acclimatization to cold. Based on those studies, cold acclimatization could be divided into the following categories: (a) insulative acclimatization (a reduction in heat loss during cold stress), (b) metabolic acclimatization (an increase in resting or basal metabolic rate (BMR)), (c) vasomotor acclimatization (a decreased susceptibility to cold pain or cold injury in the extremities), and (d) perceptual habituation (a decreased cutaneous sensory threshold for cold thermogenesis). The insulative acclimatization to cold is featured with more lowering of skin temperature. Lower skin temperatures and decreased metabolic rates were found in a group of men who stayed in Antarctica for 1 year [19]. Australian aborigines faced with the combined stress of cold and food shortage show a combined type, hypothermic-insulative adaptation. The aborigines compared to white subjects tolerated a greater lowering of the skin (*T*_sk_) and rectal (*T*_re_) temperatures and smaller metabolism related to cold [20, 21]. A metabolic cold acclimatization exists whereby a more pronounced increase in heat production is elicited by cold exposure. NST is stimulated by noradrenaline in rodents, and humans have an increased sensitivity to noradrenaline after acclimatization to cold [22]. Davis [23] found a decrease in shivering and an increase in NST from experiments with 36 subjects dressed only in shorts and exposed for 4 weeks to air temperatures of 5 to 11 °C. Local adaptation in the fingers or toes to severe cold is manifested in an acceleration of the CIVD reaction. Nomadic Lapp shepherds from Norway exhibited an earlier onset of CIVD and less pain sensation than white controls while immersing their hands in ice water for 15 min [24, 25]. Eskimos maintained higher hand temperatures and showed greater increase in finger blood flow than European subjects during hand immersion tests in 5–10 °C water [26, 27].

Dr. Suk-Ki Hong and his colleagues extensively studied the pattern of cold acclimatization of haenyeos, who wore thin cotton swimsuits even in the middle of cold winter in the 1970s. Firstly, their studies on haenyeos found evidence that chronic exposure to cold increased the metabolic rate. The BMR of haenyeos in winter, when they were diving in very cold water, was significantly elevated above values observed in summer which suggested a manifestation of a metabolic acclimatization to cold stress [28]. The increase in BMR could be due to an increased utilization of thyroid hormones [29] or to a slight increase in sensitivity to norepinephrine [22]. The metabolic acclimatization of haenyeos was in contrast to those of Eskimo or Australian aborigines living in cold climates. The differences among the ethnic groups could be explained by differences in the degree of cold stress and diet experienced by each population. It is considered that when sufficiently exposed to severe cold, humans adapt to the cold through increased metabolic rates and with an attendant increase in peripheral temperature. The second important cold-adaptive response of haenyeos was an increase in peripheral body insulation. Haenyeos’ tissue insulation was greater than that of Eskimos and Andean Indians [30]. Even though haenyeos are relatively lean individuals than non-divers in Korea, they lost less heat during cold exposure than non-divers with the same thickness of subcutaneous fat. This could be explained by the control of peripheral blood flow to the limbs. The greater peripheral insulation of haenyeos was also attributed to lower shivering thresholds than those of non-divers. Water temperature at which 50% of the haenyeos shivered was 28.2 °C, but 29.9 °C for non-divers and 31.1 °C for Korean adult males [29]. Because haenyeos showed an increase in blood flow to the limbs with no increase in heat loss [31], it was suggested that the greater insulation was due to a more efficient countercurrent heat exchange system [22, 29, 31]. The third feature of cold acclimatization was a strong vasoconstriction in the most distal part of the extremities in cold. Haenyeos’ finger blood flow and skin temperature during hand immersion in 6 °C were lower than those in non-divers [32], which is contrary to the attenuation of finger vasoconstriction in arctic fishermen or Eskimos. The difference could be attributed to the whole-body cold exposure of haenyeos compared to the local cold exposure experienced by Eskimos and arctic fishermen.

### Loss of cold acclimatization and haenyeos

Deacclimatization to the cold for haenyeos began to be reported after haenyeos started wearing wetsuits around the mid-1970s to avoid severe cold stress during diving work. As a result of the reduced cold stress, seasonal changes in metabolic rate disappeared and no difference in BMR was found between haenyeos and non-divers [27]. A decrease in *T*_re_ during diving was 2.2 °C for haenyeos wearing cotton swimsuits, but only 0.6 °C with wetsuits in winter [27]. Heat loss was reduced to 37% of what it was when haenyeos wore cotton swimsuits, but mean skin temperature was as much as 10 °C higher while wearing wetsuits [27]. The difference in critical water temperature for shivering between haenyeos and non-divers was as much as 4 °C in the 1960s but no difference was reported in 1983. Finger temperature and blood flow during hand immersion in 6 °C water among haenyeos wearing wetsuits were similar to those of non-divers, which suggests that vascular acclimatization to cold observed among haenyeos in the 1960s disappeared in the haenyeos wearing wetsuits. Park and Hong [33] suggested that lower finger temperatures for haenyeos wearing cotton swimsuits was the result of overall cold body stress rather than local cold stress to the hands because similar responses occurred with the Gaspe fishermen. Greater vasoconstriction in finger blood vessels for haenyeos wearing cotton bathing suits during cold-water immersion was sustained until the third year of wetsuit diving, but disappeared during the subsequent 3 years [4]. The adoption of wetsuits by haenyeos around the mid-1970s led to a progressive deacclimatization to cold over the next several years. The insulative acclimatization of the peripheral tissue disappeared faster than the metabolic mechanism of shivering attenuation.

## Overall cold tolerance and aging

Since the loss of cold acclimatization was reported, thermophysiological interests on haenyeos have waned. As noted in the introduction, however, diving hours have been prolonged due to the insulative wetsuits, which induced the elongation of the cold exposure of the face and hands. Haenyeos became older and 88% of haenyeos are over their sixties at present. In this light, we revisited a series of issues on older haenyeos’ physiological, behavioral, and perceptual characteristics. In this section, we introduce our results on the general cold tolerance of older haenyeos based on questionnaire studies and cold tolerance tests in a climate chamber.

**Fig. 2:**
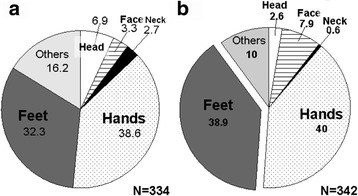
The coldest body region while diving in the sea (**a**) and when coming out from the sea after diving (**b**). Adapted from Lee et al. [6] [unit:%]

Firstly, we randomly recruited 289 haenyeos who are currently working (66 ± 8 years in age, 57.3 ± 8.1 kg in body mass, 157.1 ± 4.6 cm in height, and 54 ± 10 years in diving work career) for a questionnaire study in Jeju [6]. Seventy-nine percent of the haenyeos began breath-hold diving in their teenage years, and they still do breath-hold diving more than 10 days a month (70%) and more than 3 h a day (87%). The 87% of the respondents used to wear the thin cotton swimsuits when they were young. They recollected wearing heavier clothing in recent winters when compared to those in their twenties (85%) and most of them wear thermal underwear in winter (94%). It is, however, of interest to introduce the following results from the questionnaire. Older haenyeos felt colder in recent winter seasons (78%), and they were more sensitive to cold sea water when compared to those in their twenties (86%), whereas they considered themselves being less vulnerable (or less sensitive) to cold when compared to ordinary persons in their similar age group (68%). The coldest body regions while diving in the sea were the hands (39%) followed by the feet (32%) and head/face/neck (13%). They also felt greater coldness at those body regions when coming out from the sea after completing their diving (the hands 40%, feet 39%, and head/face/neck 11%) (Fig. 2). To sum up, older haenyeos wear much heavier clothing and feel colder lately compared to when they were in their twenties, which could be related to the effect of aging (e.g., loss of muscle mass). However, it is of interest that they recognize themselves as being less vulnerable to cold than ordinary older people which could be interpreted as perceptual-cold adaptation due to their cold-water diving. Further, our hypothesis that the overall cold-adaptive mode in their youth might switch over to local cold adaptation is supported by the fact that older haenyeos are still exposed to cold on the hands, feet, and face while diving even though they have been wearing wetsuits.

**Fig. 3:**
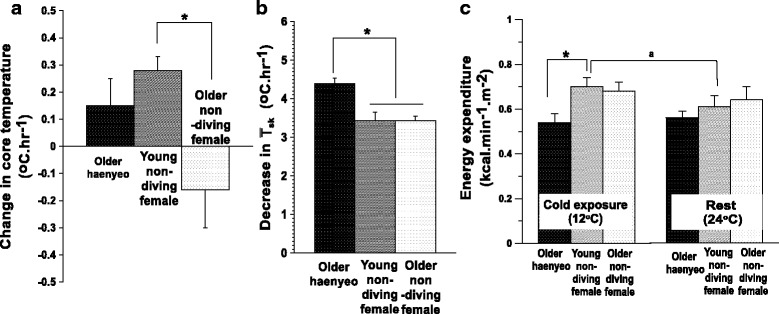
Changes in core temperature (**a**), mean skin temperature (**b**) and energy expenditure (**c**) of older haenyeos and non-diving females during cold air exposure. Adapted from Park et al. [34]

In line with the questionnaire study, we conducted overall cold tolerance tests with older haenyeos in a climate chamber. Older haenyeos (*N* = 10, 70 ± 3 years in age), young non-diving females (*N* = 10, 23 ± 2 years), and older non-diving females (*N* = 6, 73 ± 4 years) participated in the whole-body cold tolerance study. For the test of overall cold tolerance, subjects wearing light clothing were exposed to an air temperature of 12 °C for 60 min. We found that (1) older haenyeos had a greater decrease in mean skin temperatures during cold exposure while their core temperature had no statistical difference when compared to the older non-diving or young non-diving females (*P* < 0.05, Fig. 3a, b); and (2) older haenyeos had a lower metabolic rate during cold exposure than the young non-diving females (*P* < 0.05, Fig. 3c) [34]. The metabolic rate of older haenyeos being the lowest among the three groups was not the result of aging itself, but by the effect of cold-water diving over half a century. Further, we reaffirmed that older haenyeos showed less frequent shivering than the older non-diving and young non-diving females [34]. In general, shivering for older people is reduced compared to young adults or may even be absent in cold [35], but the present results are not explained by aging only, because there was a difference between older haenyeos and older non-diving females. Therefore, less active shivering would be explained by attenuation by cold habituation rather than aging. In the overall cold tolerance test, older haenyeos felt cooler on the face with lower face temperature when compared with older non-diving females and this result will be discussed in the next section on local cold adaptation. To sum up, more stable core temperature, lower mean skin temperature, and smaller energy metabolism of older haenyeos during cold air exposure indicate that older haenyeos retain general cold-adaptive traits which could be interpreted as the insulative type of cold acclimatization, but certain cold-adaptive traits were overridden by aging.

## Local cold tolerance and aging

As mentioned in Fig. 2, the coldest body regions while diving in the sea water were the hands, feet, and head/face/neck. Older haenyeos felt greater coldness when coming out from the sea after diving in winter. These results indicated that despite converting from thin cotton suits to wetsuits, older haenyeos have still been exposing their extremities to cold sea water with an insufficient amount of thermal insulation. In other words, it could be that the cold stress of haenyeos has switched from whole-body stress to local body stress. Repeated cold exposure of the local body possibly induces physiological and psychological changes that reduce distress and discomfort. Launay and Savourey [36] in their review found that the physiological features of local cold adaptation were expressed in higher skin temperature, less vasoconstriction, less pain, and an earlier cold-induced vasodilation (CIVD). Wakabayashi and his colleagues (2017) found that repeated forearm cooling for 3 weeks induced increases in oxidative metabolism in skeletal muscle metabolism which may facilitate the performance of the muscle in the cold [18]. Along with these previous findings, we introduce physiological and psychological responses of older haenyeos and discuss as strong evidence of local cold adaptation.

**Fig. 4:**
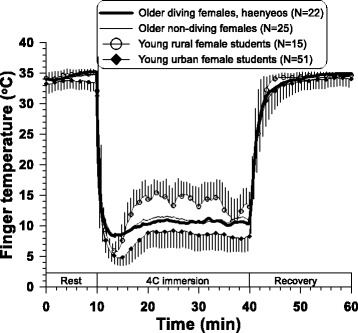
Time courses of finger temperatures during the 60-min finger immersion test at 4 °C water. Adapted from Lee et al. [37]

Firstly, we investigated the local cold tolerance of older haenyeos (*N* = 22) through finger cold-induced vasodilation (CIVD) during finger immersion in 4 °C water [37]. Older non-diving females (*N* = 25) and young non-diving females from a rural area (*N* = 15) and an urban area (*N* = 51) participated as control groups for the haenyeos. As a result of the test, evidence of both local cold adaptation and aging were found. Older haenyeos showed more pronounced CIVD responses during the finger cold immersion than older non-diving females, but such differences were limited to minimum finger temperature (*T*_min_) during cold immersion and finger temperature in recovery (*T*_recovery_). Time variables, such as onset time and peak time, showed no differences among the four groups (Fig. 4). Kang and colleagues [9] and Park and colleagues [4] reported that cold-adaptive traits of haenyeos gradually disappeared due to their thick wetsuits, but we verified that local cold adaptation for older haenyeos was found even though they have worn wet suits since the late 1970s. It is not clear whether the local cold-adaptive traits were the remnants of the general cold adaptation or a mode switch from the general adaptation to local adaptation. In Paik and colleagues’ study [34], finger temperature during hand cold immersion in 6 °C water was lower for haenyeos than non-divers. When it is associated with an isolative or hypothermic cold adaptation, the CIVD is generally reduced [32]. Greater *T*_min_ or *T*_recovery_ of the older haenyeos than older non-diving females are beneficial to prevent cold injuries on the extremities, but may not be beneficial to reduce heat loss from the extremities and prevent hypothermia in the cold. Keeping the hands warm by wearing gloves in cold water increased the overall rate of body heat loss [38].

An interesting finding in our recent studies is that the cold-adaptive traits were characterized only in temperature variables (*T*_min_ and *T*_recovery_, not *T*_max_) but not in time variables (onset time of CIVD and peak time of finger temperature) (Fig. 4). Typically, greater cold tolerance on the hand is evaluated as higher *T*_min_, *T*_max_, *T*_mean_, *T*_recovery_, and CIVD-frequencies and faster onset time (*t*_onset_) and peak time (*t*_peak_) [39]. However, we found a separated tendency in temperature variables (*T*_min_ and *T*_recovery_) and time variables (*t*_onset_ and *t*_peak_). Older haenyeos kept local cold-adaptive properties in vasoconstriction to cold exposure and vasodilation in recovery, but local cold-adaptive properties were blunted in vascular reaction velocity due to aging. Collins and colleagues [40] reported both age-related blunted and slower responses in hand vasoconstriction to cold air exposure for older men. The stiffness in skin vessels are mainly considered to be caused by age-dependent structural changes [41]. Aging is manifested in a diminished autonomic function, and recovery time after cooling was less rapid in the hands and feet of older subjects compared to young subjects [42]. The present results suggest that vasomotor reaction velocity, rather than the magnitude of vascular responses, is more under the control of autonomic function which is diminished by aging. In addition, enhancement of CIVD responses with exercise training [43] could be another reason for the pronounced CIVD responses in temperature variables for older haenyeos.

**Fig. 5:**
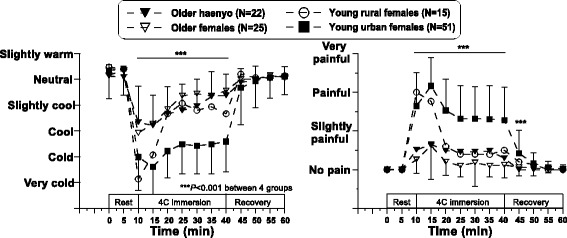
Time courses of finger thermal sensation and pain sensation during the 60-min finger immersion test at 4 °C water. Adapted from Lee et al. [37]

Habituation to cold, the most common cold adaptation, is characterized by blunted thermal sensation as well as a blunted shivering or blunted cutaneous vasoconstrictor response. Aging is characterized as a deteriorated ability to maintain homeostasis [44], and older subjects displayed reduced thermal sensitivity and expressed feelings of being in less discomfort than young subjects when exposed to cold environments [40, 44–47]. Tochihara and colleagues [48] found aging-related deterioration in cutaneous warm sensitivity on the hand and foot in cool environments. Temperature discrimination on the finger for younger subjects is approximately 0.5 °C for both coolness and warmth, increasing to 1.0–5.0 °C for the elderly [49]. In the present results, both older haenyeos and older non-diving females felt less cold and less discomfort when compared to young females (Fig. 5). This is in line with the previous studies. Another important issue is there were no differences between older haenyeos and older non-diving females. As discussed, both chronic cold exposure and aging could induce the blunted cutaneous sensitivity to cold. In this sense, older haenyeos might feel much less cold and pain from cold than older non-diving females, but no differences were found between the two older groups. We could not find any additive effect by the mixing of age-related bluntness and cold habituation on thermal and pain sensation.

**Fig. 6:**
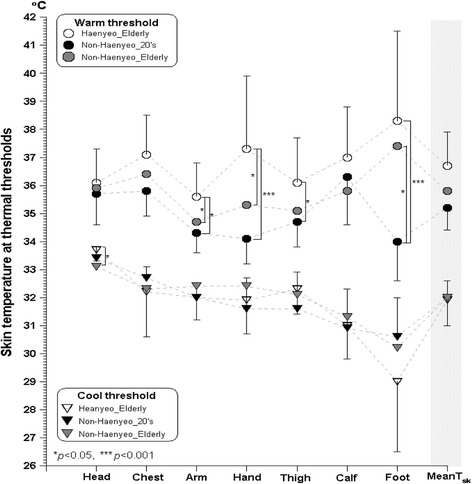
Regional skin temperatures at the moment of warm and cool thresholds for older haenyeo (*N* = 14), older non-haenyeo females (*N* = 8), and young non-haenyeo females (*N* = 10). Adapted from Kim et al. [51]

Cutaneous sensory habituation is a lesser known area compared to cardiovascular changes after cold adaptation. Theoretically, habituation is related to a function of the central nervous system (CNS) so that a threshold is altered to an unchanging stimulus, which possibly contributes to alleviate the strain or discomfort. Brück and colleagues [50] reported a reduction in thermal discomfort and cold sensation along with shifted metabolic reaction, shivering threshold, and lowered esophageal temperature by transient cold exposure from 28 to ±5 °C within 2 weeks. However, there is a lot to be revealed in this area. In particular, the reduced thermal sensitivity against cold stimulus was not clearly supported by a recent study which measured haenyeo’s cutaneous thermal threshold. We compared cutaneous warm and cool perception thresholds of older haenyeos (*N* = 14) with older non-haenyeo females (*N* = 8) and young non-haenyeo females (*N* = 10). As a result, the cool sensation threshold on the forehead was significantly greater for older haenyeos than older non-haenyeo females (Fig. 6) [51]. When combining the results of both studies mentioned above, beneficial change in thermal perception possibly could be primarily induced by the difference in initial skin temperature. On the other hand, it could also be considered that the discrepancy in thermal sensation is reinforced in the greater cold stimulus. The difference in cold sensation was greater as cold exposure was prolonged [50]. Unlike the results of cool perception thresholds, we found the significant difference in cutaneous warm sensation thresholds (Fig. 6) [51], and older haenyeos had greater warm perception thresholds on the extremities compared to older non-diving females or young females. It is difficult to explain the greater warm thresholds on the hand and feet of older haenyeos by local cold adaptation, because such great warm perception thresholds are found from heat-acclimatized people such as tropical indigenes [52]. There might be a possibility of cross-adaptation between cutaneous warm and cold perception, or other physical factors could be involved in the habituation of warm thresholds on the hand and foot of older haenyeos. More investigations are needed.

**Fig. 7:**
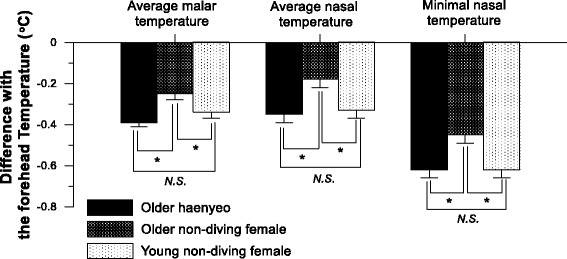
Regional differences with the forehead infrared temperature of the average malar temperature, average nasal temperature, and minimal nasal temperature for older haenyeo (*N* = 50), older non-diving females (*N* = 53), and young non-diving females (*N* = 43). Adapted from Kim et al. [53]

Facial skin temperature was another newly explored variable used to evaluate thermophysiological traits of being chronically exposed to cold sea water. Kim and Lee [53] reported distributional differences in facial temperature of older haenyeos (*N* = 50), older non-haenyeo (*N* = 53), and young non-haenyeo females (*N* = 43). The results represented the significant difference in the temperature gradient of average malar, average nasal, and minimal nasal temperature with the forehead temperature between older haenyeos and older non-haenyeos (Fig. 7). The face has the unique characteristics of being the most frequently exposed body region to external air and sea water environments, the most accessible area to be measured with infrared thermography, and where the various kinds of cutaneous vessels are located which facilitates numerous distributional appearances according to within and between subjects. Aging impairs thermoregulatory cutaneous vasoconstriction in the cold in various ways. Thompson-Torgerson and colleagues [54] explained that the impaired vascular response of the aged is due to changes in sympathetic reflex vasoconstriction, where the efferent sympathetic signal, release of norepinephrine, cotransmitters, and adrenergic sensitivity are significantly attenuated with aging. In addition, the effects of aging on locally driven vasoconstriction are related to intracellular pathways [54]. Further research is encouraged to explore the factors involved in the relatively lower malar and nasal temperature in older haenyeo’s face.

## Cross-adaptation between cold stress and heat tolerance

Older haenyeos have been exposed to cold in the following sequential routes: (1) extreme but daily short-term cold exposure of the whole body while wearing thin cotton bathing suits (until the late 1970s) and (2) mild but daily long-term cold exposure of the body extremities while wearing thick wetsuits (the early 1980s–present). As mentioned in the previous parts of this review, haenyeos have been investigated in terms of cold acclimatization (period of cotton bathing suit) and deacclimatization (period of wetsuit). In the series of our studies with older haenyeos wearing wetsuits, we found that older haenyeos’ responses to heat and cold differed from older non-diving females and young females. To explain the unexpected phenomenon, it needs to introduce the concept of cross-adaptation. Long-term exposure to a certain environment, whether continuous or intermittent, can improve tolerance to the particular environment but also affect tolerance to other adverse factors. This concept is called “cross-adaptation.” Cross-adaptation in another way can be developed as either negative or positive cross-adaptation [55]. Very few studies on humans’ cross-adaptation between cold and heat exposure have been found. According to Hessemer and colleagues [56], heat acclimation through five consecutive days at the air temperature of 55 °C for 1 h per day lowered the threshold of shivering and sweating responses, which indicates that cross-adaptation could be produced between heat and cold stresses. Lunt and colleagues reported generic autonomic cross-adaptive effects between cold adaptation and acute hypoxia-exposure in humans showing that subjects who had been exposed to cold water had a lower level of catecholamine concentration in hypoxic exposure [57].

**Fig. 8:**
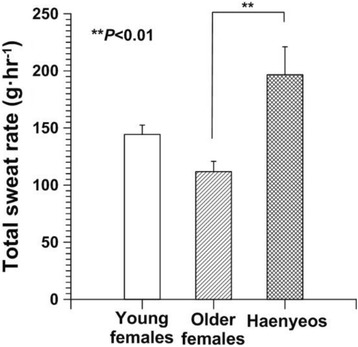
Total sweat rate (TSR) of young females (non-divers, *N* = 10), older females (non-divers, *N* = 8), and older haenyeos (*N* = 10) during the hot water-leg immersion

In this light, we explored the heat tolerance for older haenyeos with the concept of cross-adaptation. Older haenyeos (*N* = 10, 75.1 ± 3.5 years in age) were compared to older non-diving females (*N* = 8, 70.8 ± 3.8 years). Young non-diving females (*N* = 10, 23.4 ± 1.9 years) participated to investigate the effect of aging. Heat tolerance was tested during a hot water-leg immersion into 42 °C water for 60 min. We found that older haenyeos had a greater total sweat rate (196.6 ± 24.5 g · h^−1^) than older females (111.8 ± 9.1 g · h^−1^) or young females (144.3 ± 8.2 g · h^−1^) (*P* < 0.05, Fig. 8). The density of activated sweat glands on the forehead was greater in older haenyeos than in older females or young females. However, *T*_re_ and *T*_sk_ showed no significant differences among the three groups. More active sweating responses of older haenyeos than other groups imply that seawater diving at the water temperature of 10–25 °C over the past 50 years improved their heat tolerance, which might be interpreted as a cross-adaptation. This mechanism needs to be elucidated in further studies.

## Circadian rhythms in body temperature and cardiovascular responses

**Fig. 9:**
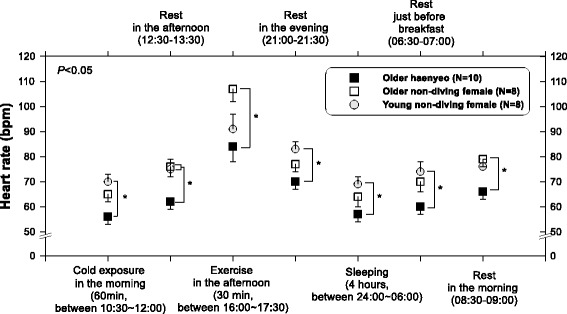
Circadian rhythm of heart rate in daily-life scenario. Adapted from Park et al. [58]

There were relatively few reports on the circadian rhythm of body temperature for cold acclimatized groups. In a recent study, we investigated older haenyeos’ circadian rhythms in body temperature and heart rate [58]. The ranges of core body temperature in older haenyeos for a day were about 1 °C (36.7–37.8 °C), and similar ranges were observed for young (36.7–37.6 °C) and older (36.8–37.9 °C) non-diving females. There were no significant differences in the changes of core temperature between the three groups, but group differences were found during the cold exposure phase which was scheduled in the circadian experiment (*P* < 0.05). Interestingly, we found that older haenyeos had lower heart rates during a 24-h period when compared to young and older non-diving female groups (*P* < 0.05, Fig. 9), whereas a previous study reported that there was no difference in the baseline value of heart rate between breath-hold divers and non-divers [59]. The differences between Dujic and colleagues’ study [59] and our study would be in regards to participants’ age (twenties vs. seventies), work experience, and sex (male vs. female). In particular, participants’ diving work experience in Dujic and colleagues’ study [59] was much shorter when compared to the older haenyeos with diving careers of over 50 years.

**Fig. 10:**
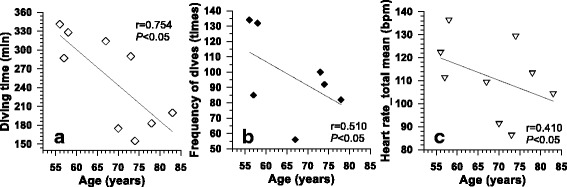
Relationships between age and total diving time (**a**), frequency of dives (**b**), and heart rate at work (**c**). Adapted from Lee et al. [60]

Regarding the lower heart rate of the older haenyeos compared to older or young non-diving females, we introduce a field study that explored diving patterns and heart rate of older haenyeos while breath-hold diving in cold seawater [60]. Cardiovascular functions of older haenyeos have become adapted to underwater environments through repetitive and chronic breath-hold diving over about 50 years. In the field study, nine haenyeos (68 ± 10 years in age, 56–83 years) were observed at a seawater temperature of 10 to 13 °C in winter. We found that older haenyeos among the nine haenyeos had lower heart rate at work (*P* < 0.05, Fig. 10), and all haenyeos showed heart rates at lower levels than typically the known heart rate levels of breath-hold divers or non-divers. Further, all haenyeos showed diving bradycardia with a decreased rate of 20 ± 8% at the bottom time (101 ± 20 bpm) when compared to surface swimming time (125 ± 16 bpm) in the sea. This could be comparable to Hong et al. (1967) found that haenyeos’ heart rate decreased from 101 bpm at rest to 60 bpm at 40 s after water immersion. It is known that oxygen consumption while breath-hold diving is minimized by selective blood circulation which flows to the brain, heart, and other necessary tissues along with blocking the flow to the intestines, kidney, or muscles through the autonomic reflex [61, 62], and simultaneously, it was reported that lung capacity was 12% greater for haenyeo (3.4 L) than non-diving women (3.1 L) [63]. The autonomic reflex and greater lung capacity along with advancing age are regarded as a complex adaptation of the cardiovascular responses of older haenyeos.

## Conclusions

For haenyeos, diving hours have been prolonged due to the insulative wetsuits, which induced the elongation of the mild cold exposure of the face and hands. It has been known that cold-adaptive traits of haenyeos disappeared but we confirmed that cold-adaptive traits are still retained to some extent. During overall cold air exposure, more stable core temperature, lower mean skin temperature and smaller energy metabolic rate of older haenyeos indicate that older haenyeos retain general cold-adaptive traits. That could be interpreted as the insulative type of cold acclimatization, but certain cold-adaptive traits were overridden by aging. The cold stress of haenyeos has switched to local mild and long-term body stress. More pronounced cold-induced vasodilation responses during the finger cold immersion was found for older haenyeors than older non-diving females, but the cold-adaptive traits were characterized only in temperature variables (*T*_min_ and *T*_recovery_, not *T*_max_) but not in time variables (onset time of CIVD and peak time of finger temperature). It is interesting such a separated tendency in temperature variables (*T*_min_ and *T*_recovery_) and time variables (*t*_onset_ and *t*_peak_). Further, we explored the heat tolerance for older haenyeos with the concept of cross-adaptation. The density of activated sweat glands was greater in older haenyeos than in older females or young females. Also, we found that older haenyeos had greater warm perception thresholds on the extremities compared to other groups. These findings imply that seawater diving at the water temperature of 10–25 °C over the past 50 years improved their heat tolerance as well as local cold tolerance, which might be interpreted as a positive cross-adaptation. Further studies on cross-adaptation between cold stress and heat tolerance are needed.

## Abbreviations

BAT: Brown adipose tissue
BMR: Basal metabolic rate
CIVD: Cold-induced vasodilation
NST: Non-shivering thermogenesis
*T*_sk_: Skin temperature
*T*_re_: Rectal temperature
*T*_max_: Maximum temperature
*T*_mean_: Average temperature during immersion
*T*_min_: Minimum temperature
*T*_recovery_: Temperature in recovery
